# From thought to language: Comparing schizophrenia spectrum disorders and Wernicke’s aphasia with machine learning and LLMs

**DOI:** 10.1016/j.scog.2026.100443

**Published:** 2026-05-26

**Authors:** Perry van der Zande, Andreas van Cranenburgh, Frank Tsiwah

**Affiliations:** University of Groningen Center for Language and Cognition, Netherlands

**Keywords:** Schizophrenia spectrum disorders, Wernicke’s aphasia, Language and thought, Machine learning, LLMs

## Abstract

Schizophrenia spectrum disorders (SSD) and Wernicke’s aphasia (WA) both disrupt meaningful speech, yet they arise from fundamentally different disturbances in thought and language. SSD is defined by formal thought disorder, in which disorganized thinking is inferred from abnormalities in speech, whereas WA reflects a primary breakdown of language implementation following focal brain damage. We investigated whether quantitative markers of lexical–semantic, syntactic structure, and semantic coherence in spontaneous speech can distinguish SSD, WA, and healthy controls.

Using Natural Language Processing techniques, we extracted syntactic, lexical and local semantic similarity features from spontaneous speech transcripts and used them in supervised machine learning models to classify diagnostic groups. In parallel, an instruction-tuned large language model (LLM) was used in a zero-shot setting to assign transcripts to diagnostic categories and to track the severity of language disturbance.

Our results showed a distinct linguistic pattern, particularly in syntactic and local semantic organization for WA, indicating a paradigmatic language disorder. By contrast, the same features were less effective in distinguishing SSD from matched controls, in line with the view that SSD reflects a more diffuse disturbance of thought that only partially manifests in surface language. Zero-shot LLM classifications approached the performance of supervised models for WA-related contrasts and were sensitive to graded language disturbance. At the same time, strong task and dataset effects underscored the need for carefully controlled speech elicitation. Together, these findings highlight both the promise and the limitations of automated language analysis for clinical diagnostics and for understanding speech and thought abnormalities.

## Highlights

• SSD and Wernicke’s aphasia show partially distinct quantitative linguistic profiles.
• NLP features differentiate aphasic from psychiatric language disturbance.
• Zero-shot LLM approaches supervised performance with less corpus bias.
• Task type and prompt substantially influence extracted linguistic features.

## 1 Introduction

Schizophrenia spectrum disorders (SSD) and Wernicke’s aphasia (WA) are both marked by disturbances in speech and/or language. SSD is a psychiatric condition characterized by symptoms such as hallucinations and delusions, which are sometimes accompanied by language impairments referred to as formal thought disorder (FTD; American Psychiatric Association, 2022). Linguistic features including derailment, tangentiality, and the use of neologisms have been widely described in the speech of individuals with SSD (Kuperberg, 2010, Chaika, 1982). In contrast, WA is a neurological disorder caused by focal brain damage (often due to stroke) that primarily affects language comprehension and production, frequently resulting in fluent yet nonsensical speech (Damasio, 1992a, Acharya and Wroten, 2023). Despite their distinct etiologies and clinical profiles, the language patterns in these disorders can be strikingly similar. Butler and Zeman (2005) observed that both patient groups may produce “word salads” that even experienced clinicians, such as neurologists, psychologists, and speech-language therapists, find difficult to distinguish (Faber et al., 1983). This resemblance can lead to misdiagnosis, with cases of WA erroneously identified as psychosis, particularly in the absence of neuroimaging, thereby delaying timely stroke treatment (Lane et al., 2011, Sambunaris and Hyde, 1994, Faber et al., 1983).

The advent of natural language processing (NLP) and other machine learning (ML) techniques, with their enhanced sensitivity to subtle patterns in language data, has opened new avenues for objectively quantifying and characterizing speech and language disturbances. For instance, Tang et al. (2021) demonstrated that NLP-based methods could successfully identify linguistic differences between individuals with SSD and healthy controls, even when standard clinical ratings failed to distinguish the groups. Therefore, the goal of the current study is to use machine learning approaches to systematically investigate the extent to which the language characteristics of people with schizophrenia and Wernicke’s aphasia differ from, or overlap with, each other.

The intersection of language and thought is particularly salient in understanding schizophrenia spectrum disorders and Wernicke’s aphasia, as both conditions significantly disrupt meaningful verbal expression, though by distinct mechanisms (Butler and Zeman, 2005, Hinzen and Rosselló, 2015, Little et al., 2019). In SSD, FTD forms a clinical cornerstone, manifesting as disorganized thinking that is typically inferred from speech abnormalities including derailment, tangentiality, and neologism production (Kuperberg, 2010, Chaika, 1982). Importantly, contemporary research and clinical frameworks regard FTD not merely as a linguistic anomaly, but as a pragmatic marker of broader disruptions in the organization of thought. This perspective is increasingly evidenced in neuropsychiatric and linguistic research, which link FTD to core deficits in conceptual organization, abstraction, and reasoning (Çokal et al., 2018, Jerónimo et al., 2018, Marggraf et al., 2020, Nordgaard et al., 2021, Stein et al., 2025). Nevertheless, it is important to note that the boundary between thought and language is not always easily drawn, and FTD may itself encompass both conceptual and linguistic processing disruptions to varying degrees.

A growing body of research demonstrates that FTD reflects disturbances extending beyond language alone to encompass broader cognitive and organizational dysfunction. FTD has been shown to affect both linguistic and neurocognitive domains, serving as a robust marker of illness severity across psychiatric syndromes (Stein et al., 2025). Linguistic analyses further reveal that FTD correlates with impaired referential and syntactic abilities, indicating deficits in cognitive processes fundamental for coherent language use (Çokal et al., 2018). Moreover, FTD is increasingly conceptualized as a trait-like phenomenon closely linked with self-disorder and disruptions in conceptual organization (Nordgaard et al., 2021), indicating its status as a stable marker of disturbed thought. Historical and theoretical perspectives trace FTD to core deficits in organizing and abstracting thought, with observable consequences manifesting as reduced discourse coherence and impaired communicative precision (Jerónimo et al., 2018). Finally, the clinical relevance of FTD is reinforced through its association with global functional impairments and breakdowns in thought coherence observed within the schizophrenia spectrum (Marggraf et al., 2020). Altogether, these findings establish FTD as a pragmatic indicator of disrupted thought organization rather than a mere linguistic anomaly (Çokal et al., 2018, Jerónimo et al., 2018, Marggraf et al., 2020, Nordgaard et al., 2021, Stein et al., 2025).

In contrast, Wernicke’s aphasia (WA) arises from focal neurological injury, most commonly to the posterior superior temporal gyrus, Brodmann area 22, of the language-dominant hemisphere, a cortical region essential for speech comprehension and semantic integration (Damasio, 1992b, Acharya and Wroten, 2023, Turken and Dronkers, 2011, Binder et al., 2009). Damage to this area, and its connected network extending to the middle temporal and inferior parietal regions, disrupts the neural mechanisms that map auditory–verbal input to conceptual representations, resulting in severe comprehension deficits and impaired semantic access (Jefferies and Lambon Ralph, 2006, Thompson et al., 2015, Fridriksson et al., 2010). Despite relatively preserved fluency, prosody, and syntactic structure, speech in WA is often devoid of meaningful content, characterized by paraphasias, neologisms, and circumlocutions, which obscure lexical precision and coherence (Goodglass and Kaplan, 1983, Robson et al., 2012, Ardila et al., 2016). This dissociation, grammatically intact but semantically impaired speech, indicates that WA reflects a failure of the neural mechanisms linking words to concepts and contextual meaning rather than a disorder of ideation itself (Binder et al., 2009, Lambon Ralph, 2014, Turken and Dronkers, 2011). Moreover, individuals with WA frequently exhibit limited awareness (anosognosia) of their impairment, continuing to produce error-laden but fluent language without recognizing its unintelligibility (Lebrun, 1987, Jefferies and Lambon Ralph, 2006, Robson et al., 2012). Cumulatively, lesion-mapping, linguistic, and cognitive evidence position WA as a predominantly language-level disorder, where disruption to modality-specific neural networks undermines the conversion of thought into linguistic form. This contrasts with the conceptual disorganization more central to SSD, though the distinction reflects a difference in primary locus of impairment rather than a clean dissociation.

Comparative studies illustrate the difficulty in disentangling these processes. Faber et al. (1983) showed that experts often misclassified WA as SSD when reading decontextualized transcripts, as both patient groups produce ”word salad” speech marked by neologisms and incoherence. Yet more subtle distinctions emerge at the intersection of language and thought: SSD speech, though fragmented, often retains grammatical complexity and exhibits illogical or tangential connections reflecting disrupted reasoning, whereas WA speech is marked by simplified syntax, poverty of content, and pervasive word-finding difficulties, suggestive of a breakdown in the mechanisms that tie words to concepts and real-world knowledge. These observations underscore the importance of studying language not in isolation, but as a manifestation and metric of thought disorder.

The application of natural language processing (NLP) methods has thus become an indispensable tool for examining the language–thought interface in these disorders. Metrics such as semantic similarity between adjacent speech units, syntactic complexity, and lexical diversity provide measurable proxies for the underlying processes of ideation, conceptual connectivity, and linguistic encoding. Recent work using word and sentence embeddings, transformer-based models, and surprisal scores has demonstrated that specific linguistic features extracted from spontaneous speech can be reliably mapped onto clinical phenomena associated with disordered thinking and aphasic impairment (Tang et al., 2021, Jeong et al., 2023, Tsiwah et al., 2024). For example, greater sentence-level semantic divergence and elevated syntactic complexity are more aligned with SSD thought disorder, whereas reduced lexical diversity and intra-sentential semantic loss distinguish WA.

Taken together, these developments motivate a systematic, data-driven approach to investigating the specific language–thought relationships that set SSD and WA apart. The present study leverages a multidimensional suite of linguistic features, including syntactic and semantic measures, to characterize and classify spontaneous speech in both groups, and complements this feature-based approach by zero-shot prompting a large language model to assess diagnostic distinctions directly from text, without task-specific training. By anchoring analyses in foundational theories and integrating the latest computational advances, this work aims to characterize the degree of overlap and divergence between aphasic and psychiatric language disturbance, with the goal of identifying linguistic markers that are differentially, if not exclusively, associated with each condition.

## 2 Methods

### 2.1 Data and preprocessing

For aphasia we used semi-structured spontaneous speech transcripts from AphasiaBank (MacWhinney et al., 2011), which contained 34 transcripts of people with Wernicke’s aphasia (WA) and 34 healthy control transcripts (referred to as HCA), randomly selected from the *Capilouto* dataset (Capilouto and Wright, 2025) to match the WA group. The random selection was repeated three times to ensure robustness with respect to the undersampling. The transcripts used for this study were based on open-ended questions (e.g., *tell me about an important event in your life*) and picture descriptions tasks (e.g., *a cat rescue*). All of the WA patients have been assessed using the Western Aphasia Battery (WAB), with a mean aphasia quotient of 51.8 (SD = 13.6, range: 28.2–74.4)

For SSD we used a dataset provided by Tang et al. (2021). This data contains spontaneous speech transcripts of 27 people with SSD and 37 healthy controls (HCS), of which 27 have been randomly selected to match the SSD group size. It is important to note that the current dataset is an updated version of the dataset used in Tang et al. (2021), where different interview questions were used for cohorts 1 and 2. Following Tang et al. (2021), we treat the current dataset as an expanded version of cohort 1, as none of the interview questions used for cohort 2 appear in our transcripts. Both the SSD and the HCS groups had been evaluated using the Scale for the Assessment of Thought, Language, and Communication (TLC; Andreasen, 1986), with a mean TLC global score of 1.10 (SD=0.939, range: 0-3) for the SSD group and a mean of 0.108 (SD=0.315, range: 0-1) for the controls.

A summary of the demographic characteristics of the 4 groups can be found in Table 1. We used only the participants’ speech output, and thus all words by the interviewers were removed. All data were pre-processed, and fillers or any symbols annotated in the speech transcripts were removed. Word repetitions were excluded prior to feature extraction to avoid artificially inflating dependency and similarity scores; however, given their potential clinical relevance, repetition frequency was additionally retained as a separate feature and evaluated in combination with the syntactic and semantic sets (see Section 3.1). Incomplete words were filtered out and used for separate analysis. The SSD transcripts were obtained from Tang et al. (2021) with authorization from the corresponding author; these data are not publicly available (access inquiries may be directed to that team). The WA transcripts were accessed through AphasiaBank, which is available to registered members of the AphasiaBank Consortium.

**Table 1:** Summary of demographic and transcript data. These values correspond to the full dataset (i.e., not undersampled). For age, education level, and word count, mean values are reported. Word count refers to the total number of words uttered by the participant, aggregated over the whole transcript or all open-ended speech tasks.

Cohort	AphasiaBank		Current dataset based on Tang et al. (2021)		Tang et al. (2021)			
	HCA	WA	SSD	HCS	SSD		HCS	
					1	2	1	2
*n*	72	33	29	37	15	5	5	6
# Male	42	25	17	17	11		4	
# Female	30	8	12	20	9		7	
Age (years)	76.3	66.3	24.4	30.1	36.5		35.6	
Age range	53–89	40–81	19–37	21–38	–		–	
Education level (years)	–	–	13.6	16.8	13.4		15.8	
Word count (all tasks)	1680	1148	426	716	–		–	
Word count (open-ended)	904	595	214	363	1782		1748	

### 2.2 Feature extraction

Features were extracted separately for each task instance (open-ended questions and picture description) in a transcript. Consequently, all features were computed on participant-level by taking the mean of the feature values across all tasks within a transcript, weighed by the length of the transcript. This allows for classification on participant-level.

#### 2.2.1 Syntactic features

To automatically extract syntactic features from the transcripts, the open-source Python script UDstyle (van Cranenburgh, 2022) was used. This script parses a given piece of text using the Stanza package (Qi et al., 2020), creating dependency trees and assigning part-of-speech tags for every sentence, following the Universal Dependency formalism (UD; de Marneffe et al., 2021). Based on Stanza’s output, UDstyle calculates 9 (morpho-)syntactic complexity metrics, namely, mean number of words per sentence (LEN), Lexical density (LXD), Mean dependency distance (MDD), Normalized dependency distance (NDD), Proportion of adjacent dependencies (ADJD), Left Dependency direction (LEFT), Frequency of nominal modifiers (MOD), Mean number of clauses per sentence (CLS), and Average clause length (CLL).

In addition to the complexity metrics, UDstyle also outputs the frequency of all dependencies. Specifically, it uses the 63 dependency relations (including subtypes) defined for the Universal Dependency formalism^1^ The part-of-speech (POS) tag frequencies are outputted as well.^2^ So in total, UDstyle provides three sets of features that are partly syntactic and partly lexical in nature; for simplicity, they will be grouped under ‘syntactic’ features.

#### 2.2.2 Semantic features

To extract semantic features, word and sentence embeddings were extracted from the transcripts of the participants. The cosine similarity of the embeddings was calculated to create a total of 8 semantic features, as explained below. These are meant to capture the local and global semantic similarity of speech over varying distances, which have been used as proxies for coherence and tangentiality (e.g., Tsiwah et al., 2024, Voppel et al., 2023), though the relationship between such measures and human-rated coherence is not straightforward (He et al., 2026).

##### Embeddings and cosine similarity.

**Fig. 1:**
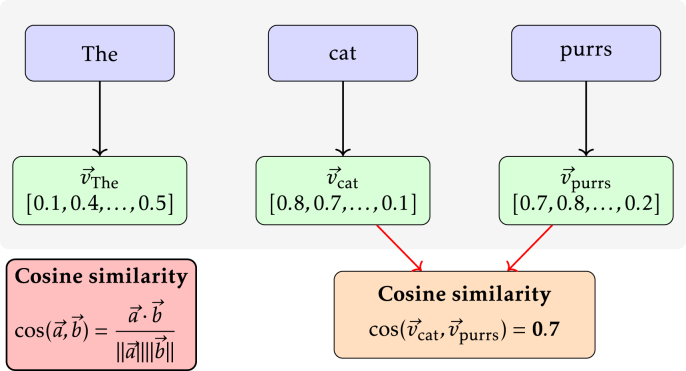
An example illustrating the extraction of cosine similarity of consecutive words.

Word embeddings are numerical representations (vectors) of words. They intend to capture word meaning based on the context in which words appear. Word embedding models capture linguistic patterns in large text corpora. Words that have similar meanings usually have similar vectors. The similarity of two given vectors can be measured by calculating the cosine of their angle, which is called the cosine similarity. It provides a convenient similarity score between −1 and 1, with -1 meaning perfectly opposite, 0 no similarity, and 1 meaning perfectly similar. The concept has also been extended to sentence embeddings. In this study, we used a pre-trained fastText model to extract word embeddings (Mikolov et al., 2018). Similarly to the word2vec model used by Tsiwah et al. (2024), word embeddings are learned from a corpus by trying to predict target words based on surrounding words. For each word, the word embedding is retrieved from the fastText model. The cosine similarity between word embeddings is calculated for each word and the word directly after. The similarity scores are then aggregated for each task instance into a mean and variance score. See Fig. 1 for an illustration.

In addition to word embeddings, sentence-level embeddings were derived using Sentence Transformers, a modification of BERT designed to produce fixed-size, semantically meaningful representations of sentences to which cosine similarity can be applied (Reimers and Gurevych, 2019, Devlin et al., 2019). Each transcript was first split into sentences based on sentence-ending punctuation. For each sentence, a sentence embedding was created by an SBERT model, and cosine similarity was then calculated within a moving window of 1 to 3 consecutive sentences. In this procedure, a window size of 1 compares each sentence only with its immediate successor, whereas larger windows (2 and 3) capture similarity across slightly longer local contexts within the transcript. For each window size, the mean and variance of cosine similarity values were extracted, yielding six sentence-level features per task instance.

### 2.3 Models for classification

Two complementary classification approaches were employed: a supervised “hand-crafted feature” approach, using traditional machine learning models trained on extracted linguistic features, and a zero-shot approach, using a large language model to classify transcripts directly from text. This combination allows assessment of both the diagnostic utility and interpretability of explicitly defined features, as well as the practical potential of contemporary foundation models that can generalize without task-specific training. The supervised models were evaluated at participant level, since this is most similar to what information is available for a real diagnosis, and allows for comparison with previous results. Zero-shot LLM classification was applied at task level to reduce the influence of broader participant context (e.g., illness history) and focus the model on how speech is structured within a single task. This task-based setup also avoids potential degradation of LLM performance with longer inputs (Hong et al., 2025), providing a more conservative estimate relative to the participant-level models.

#### Supervised machine learning approach using extracted features.

To assess how well the extracted linguistic features discriminate WA and SSD, we trained and tested Random Forest (RF) and Gaussian Naive Bayes (NB) classifiers on different feature set combinations, using the default implementation and hyperparameters in scikit-learn (Pedregosa et al., 2011). Both models were trained on syntactic and semantic features separately and in combination. We then evaluated on four binary classification tasks: WA vs SSD, WA vs HCA, SSD vs HCS, and HCA vs HCS, with the two control groups (HCA-HCS) compared to assess the impact of dataset-related factors (e.g., transcription protocol, demographics) on the feature sets. Further, for the cross-dataset contrasts (WA vs SSD, HCA vs HCS), only open-ended speech tasks were included, under the assumption that different open-ended prompts yield more comparable linguistic patterns than different picture description tasks (Tsiwah et al., 2024).

For each combination of feature set and classification grouping, performance was computed at participant level. Leave-one-patient-out cross-validation was then applied, using participant ID as the grouping factor. For the WA vs SSD condition, we extracted SHAP (SHapley Additive exPlanations) values (Lundberg and Lee, 2017) for the Random Forest model to identify the 10 most informative features. SHAP values were computed per fold and averaged across folds to obtain overall importance estimates, which are reported as mean absolute SHAP values. For all classification tasks involving the HCS group, results were additionally averaged across three independent random undersampling runs to obtain stable performance estimates robust to control selection variability. Accuracy was used as the primary evaluation metric, as all classification conditions use balanced group sizes through controlled undersampling, mitigating the main concern of accuracy being misleading on imbalanced datasets. Confusion matrices and per-class F1, precision, and recall scores are reported in Appendix C. For each configuration, the confusion matrix reflects the single run whose accuracy was closest to the mean across all 15 runs (three undersampling iterations ×five cross-validation runs), selected as the most representative run. In addition to the four binary classification conditions, a ternary classification was performed combining WA, SSD, and a matched HCS group into a single three-class problem, providing a more ecologically valid test of the model’s ability to jointly distinguish all three groups. A detailed implementation of all classification procedures is available in the accompanying code repository.^3^

#### Zero-shot classification.

In addition to the supervised models, we performed zero-shot classification of task instances using a locally run, open-source, instruction-tuned 24B-parameter Mistral Small 3 model (Mistral AI, 2025), prompted to assign each transcript to one of two classes for three binary tasks: WA vs SSD, WA vs HCA, and SSD vs HCS. Zero-shot prompting was preferred over fine-tuning because the dataset is relatively small and prior work has shown that prompt design critically shapes zero-shot performance (Yong et al., 2023). For each instance, the model received a two-part prompt: a system message specifying the classification instructions and a user message containing the preprocessed transcript preceded by a brief task description (e.g., “Tell me how things have been going recently”), providing contextual expectations for the speech sample (Yong et al., 2023). For each classification task, we used two system prompts: a “guided” prompt including typical speech characteristics of the target groups and an “unguided” prompt without such information. Example prompts are shown in Appendix A. The model context was reset before each prediction so that all transcripts were classified independently.

**Table 2:** Summary of the supervised learning experiments. Accuracy scores are averages over 15 runs (3 undersampling iterations × 5 leave-one-patient-out cross-validation runs). Between brackets are the standard deviations of the accuracies, taken over all 15 runs. Synt: syntactic, Sem: semantic, Rep: repetition frequency, Ternary: WA vs SSD vs HCS.

Features	WA vs SSD		WA vs HCA		SSD vs HCS		HCA vs HCS		Ternary	
	RF	NB	RF	NB	RF	NB	RF	NB	RF	NB
Synt.	91.0	83.9	94.9	91.4	64.9	63.0	96.0	91.7	66.6	67.1
	(0.023)	(0.000)	(0.025)	(0.038)	(0.034)	(0.015)	(0.021)	(0.054)	(0.033)	(0.025)
Sem.	87.5	85.7	94.0	96.5	53.5	60.5	77.3	82.2	55.0	60.2
	(0.018)	(0.000)	(0.016)	(0.007)	(0.076)	(0.031)	(0.049)	(0.016)	(0.031)	(0.020)
Synt.+sem.	91.3	85.7	96.9	93.9	62.6	60.5	97.4	92.2	66.4	67.5
	(0.018)	(0.000)	(0.020)	(0.025)	(0.038)	(0.038)	(0.018)	(0.055)	(0.033)	(0.026)
Synt.+sem.	93.0	85.7	97.4	94.4	62.3	61.1	96.4	92.2	68.1	69.5
+rep.	(0.015)	(0.000)	(0.019)	(0.031)	(0.034)	(0.030)	(0.018)	(0.055)	(0.036)	(0.032)

**Fig. 2:**
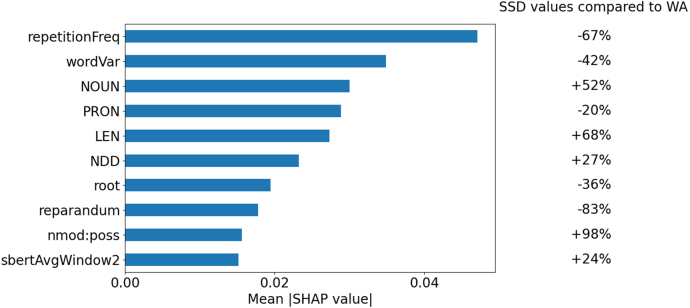
SHAP-based feature importance scores of the 10 most informative features for WA vs SSD classification. “repetitionFreq” refers to the frequency of repeated words, per 1000 words. “wordVar” and “sbertAvgWindow2” refer to the variance in word similarities and to the average sentence similarity for window size 2 respectively, as explained in Section 2.2.2. “NOUN” and “PRON” refer to the POS frequencies of nouns and pronouns. “LEN”, “NDD” and “CLS” are complexity metrics as described in Section 2.2.1. “root” and “mark” are dependency frequencies following the Universal Dependency formalism.

## 3 Results

### 3.1 Supervised machine learning using extracted features

Table 2 summarizes the supervised classification accuracies, by averaging across all tasks. For WA vs SSD, the RF-model achieved 91.0% accuracy with syntactic features and 87.5% with semantic features. Combining both feature types yielded 91.3%. Adding repetition frequency to the combined feature set further improved WA vs SSD accuracy from 91.3% to 93.0% (RF), suggesting that repetitive speech patterns capture complementary diagnostic information not fully encoded by syntactic and semantic features alone (Table 2). WA could be distinguished from HCA with very high accuracy (up to 98.5% when using NB with semantic features), indicating that both feature classes robustly capture WA-related language abnormalities. In contrast, SSD vs HCS classification was substantially low, with a maximum accuracy of 64.8% (NB, syntactic features), suggesting that the current feature sets are less sensitive to SSD-related differences. For the ternary classification (WA vs SSD vs HCS), the RF model achieved 68.1% accuracy with syntactic + semantic + repetition features, and NB achieved 69.5% (Table 2). While lower than the WA vs SSD binary accuracy, as expected given the added classification complexity, performance remained substantially above chance (33%), suggesting that the feature sets capture sufficient group-level differentiation.

#### 3.1.1 Feature importance

Fig. 2 shows that the most informative feature for WA vs SSD is the frequency of repetition followed by variance of word-level averaged cosine similarity (wordVar), which are both markedly lower for SSD than for WA. Similar group differences are observed for other semantic features, including the mean sentence-level cosine similarity with a window size of 2 (sbertAvgWindow2), which, unlike word level similarity, is lower for WA. Among the syntactic features, SSD shows higher frequencies of nouns and conjunction-related dependencies, longer mean sentence length (LEN), as well as lower pronoun frequency compared to WA. The strongest difference between the SSD and WA was the possessive nominal modifier (nmod-poss) with the former group producing 98% more than the latter group.

Feature importance scores were not computed for SSD vs HCS, because the low classification accuracies indicate that the models did not learn reliable group distinctions, making any resulting importance estimates an unstable and potentially misleading reflection of differences between SSD and healthy controls.

**Fig. 3:**
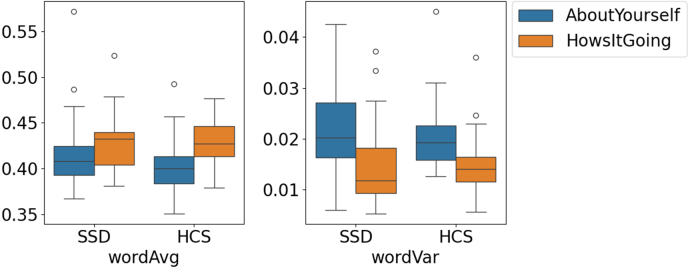
The word similarity average and variance features, per participant group and per open-ended speech task in the dataset provided by Tang et al. (2021).

#### 3.1.2 Effect of task on classification

Fig. 3 shows boxplots of the mean and variance of word-level cosine similarity for the “AboutYourself” and “HowsItGoing” tasks in SSD and HCS. Across both groups, “AboutYourself” consistently yields lower mean similarity and higher variance than “HowsItGoing”, indicating a robust task effect rather than a diagnostic effect. To quantify this, within-participant Wilcoxon signed-rank tests were performed separately for SSD and HCS, on both mean and variance, with Benjamini–Hochberg FDR correction for multiple comparisons. All four comparisons were significant (mean similarity: SSD p=0.049, HCS p=0.003; variance: SSD p=0.019, HCS p=0.001).

**Table 3:** Classification accuracies of the zero-shot classification experiments. All experiments were performed at task-level.

Prompt	Speech tasks	WA vs SSD	WA vs HCA	SSD vs HCS
Minimal	Open-ended	82.0	87.5	70.1
	Picture description	74.4	74.5	59.9
	All	79.3	79.0	63.9

Guided	Open-ended	76.3	88.0	68.2
	Picture description	69.8	84.7	63.0
	All	75.7	85.5	65.1

### 3.2 Zero-shot classification

The outcomes of the zero-shot classification experiments are summarized in Table 3. For WA vs SSD, the LLM achieved accuracies of 79.3% with the minimal prompt and 75.7% with the guided prompt, with performance consistently higher for open-ended than for picture description tasks. For WA vs HCA, accuracies were 79.0% (minimal) and 85.5% (guided), again with better performance on open-ended tasks. Performance was lowest for SSD vs HCS (63.9% minimal; 65.1% guided), although accuracies on open-ended tasks were higher than on picture descriptions for all contrasts.

**Fig. 4:**
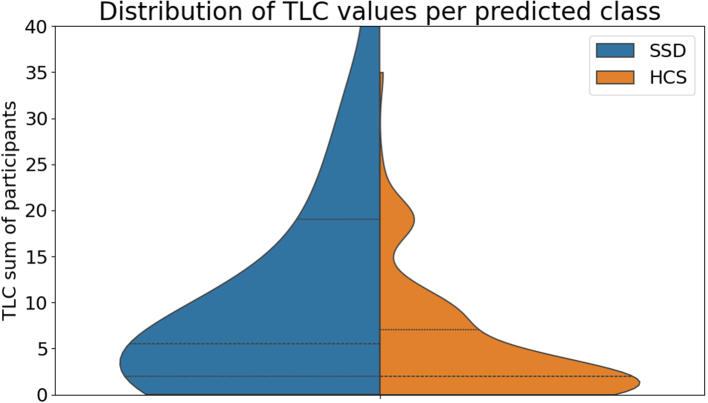
The distribution of TLC scores per class as predicted by the zero-shot classification experiment. The plot is based on classification on all tasks using the guided prompt.

To examine whether the model’s SSD predictions were sensitive to language disorder severity, Fig. 4 displays violin plots of TLC sum scores for task instances predicted as SSD versus HCS by the zero-shot LLM (guided prompt, all tasks). The distribution for instances classified as SSD is shifted towards higher TLC scores, with almost all task instances from participants scoring above 20 on the TLC classified as SSD. By contrast, instances classified as HCS are concentrated at the lower end of the TLC range, with relatively few high-scoring cases in this group. This pattern indicates that the model’s zero-shot predictions are systematically related to the clinical severity of language and thought disorder as quantified by the TLC.

## 4 Discussion

This study investigated whether quantitative linguistic markers derived from spontaneous speech can distinguish schizophrenia spectrum disorders (SSD), Wernicke’s aphasia (WA), and healthy controls, using both supervised machine learning and zero-shot large language model (LLM) classification. Given that WA is typically viewed as a paradigmatic disorder of language implementation, whereas SSD is characterized as a disturbance of thought with secondary linguistic manifestations, the central question was whether these contrasting profiles would be reflected in quantitative patterns of speech organization across groups.

### 4.1 Language and thought dissociation

The high accuracies for WA versus SSD in both the supervised and zero-shot models suggest that, despite overlapping clinical characterization of “word salad” (Butler and Zeman, 2005, Hinzen and Rosselló, 2015, Little et al., 2019) the disorders may exhibit systematically different quantitative linguistic profiles, though dataset- and task-related factors may also contribute to these differences. WA was characterized by higher variance in local word similarity, lower mean sentence similarity at certain window sizes, and lower frequency of nouns but higher frequency of pronouns than SSD, suggesting more fluctuating and lexically richer but semantically unstable output. This aligns with the classical characterization of WA as fluent, syntactically well-formed speech with impaired lexical–semantic mapping, frequent paraphasias and neologisms due to the focal temporal–parietal damage (Goodglass and Kaplan, 1983, Robson et al., 2012, Turken and Dronkers, 2011).

By contrast, SSD speech in this sample showed lower variance in word similarity and comparatively higher syntactic complexity (CLS: average number of clauses per sentence, and NDD: average dependency distance), and usage of some syntactic categories (nouns, nmod:poss: possessive nominal modifier). These patterns are consistent with the view that formal thought disorder more prominently reflects disorganization of conceptual structure and inference than a primary breakdown of lexical access and syntactic mechanisms, with language abnormalities serving as an observable surface manifestation of disordered thought organization (Andreasen, 1979, Chaika, 1982, Bedi et al., 2015, Çokal et al., 2018, Jerónimo et al., 2018, Nordgaard et al., 2021, Hinzen and Rosselló, 2015). However, prior work reporting lexical–semantic disturbances in SSD, including N400 abnormalities (Kuperberg et al., 2010), indicates that such impairments are not absent. The present data therefore support a relative rather than absolute distinction from WA. The present results are therefore compatible with the theoretical distinction, grounded in lesion and clinical phenomenology, between WA as a predominantly language-level disorder (Turken and Dronkers, 2011, Thompson et al., 2015) and SSD as a condition in which thought disturbance is more central (Andreasen, 1979, Ceccherini-Nelli and Crow, 2003), though given the cross-corpus design and modest sample sizes, this interpretation remains tentative.

At the same time, the successful discrimination between WA and SSD indicates that NLP-based measures can resolve diagnostic uncertainty in precisely the situations that are clinically most challenging, for example when WA is misinterpreted as psychosis in emergency settings (Faber et al., 1983, Sambunaris and Hyde, 1994, Edelkraut et al., 2022). This extends previous evidence that semantic similarity measures can separate SSD from healthy controls (Voppel et al., 2021, Voppel et al., 2023, Tang et al., 2021), by providing preliminary evidence that these approaches may also differentiate aphasic and psychiatric language disturbance (Tsiwah et al., 2024). The ternary results further confirm this pattern. That is, the above-chance three-way classification indicates that the linguistic profiles of all three groups are partially distinguishable, albeit with reduced precision compared to pairwise contrasts.

An important caveat to this interpretation is the very high HCA vs HCS classification accuracy, which indicates that cross-corpus differences, such as demographic composition, recording conditions, and transcription conventions, substantially distinguish the two control groups. This in turn raises the question of whether the WA vs SSD separation is driven at least partly by dataset-level artefacts rather than disorder-specific linguistic profiles. For the supervised models, this confound cannot be fully ruled out. However, the zero-shot LLM results offer a stronger basis for cautious optimism: the LLM classifies individual task responses without training on the corpus and without access to distributional patterns across participants, making it considerably less susceptible to dataset-level artefacts. Its above-chance WA vs SSD accuracy therefore provides complementary evidence that the two groups exhibit genuinely different linguistic characteristics, even if the magnitude of supervised performance is inflated by cross-corpus factors.

Nevertheless, the SSD versus healthy control classification remained modest across models and feature sets, with accuracies around chance to mid-60% despite the use of lexical, syntactic and semantic features, as well as a zero-shot approach. This contrasts with earlier work reporting higher SSD–healthy control accuracies using similar methods, include word2vec-based connectedness (e.g., 85% in Voppel et al., 2021) and BERT-based POS and coherence features (e.g., 87% in Tang et al., 2021). Several methodological factors likely contribute to this discrepancy. The underperformance of guided relative to minimal prompts likely reflects a conflict between the provided diagnostic descriptions and the model’s pre-existing knowledge, or guidance that is too narrow in scope. The zero-shot approach was included as a corpus-independent complement to the supervised models rather than as a prompt optimization exercise. Future work should systematically vary prompt wording and level of diagnostic guidance to better understand how prompt design shapes LLM classification performance in clinical speech contexts.

First, SSD participants in the current dataset had generally mild language disorder, as reflected in low TLC global scores, which could translate to reduced distinctiveness of the linguistic features available to the models. Tang et al. (2021) noted that their original dataset contained a class imbalance and task differences between groups, and observed very strong effects for incomplete words that are not replicated in the updated, more balanced dataset used in this study (see Appendix B). Second, our transcripts are shorter on average than those in some prior studies, which constrains the stability of distributional measures such as window-based similarity and syntactic frequencies. Third, our analyses indicate that connectedness features are highly sensitive to the type of elicitation task: even within the same diagnostic group, different open-ended prompts induced systematic differences in mean and variance of word similarity. If diagnostic groups differ in task composition, such task effects can be mis-attributed to diagnosis, inflating apparent classification performance. Together, these factors suggest that the relatively modest SSD classification performance in this study is not simply a failure of NLP sensitivity but reflects a combination of mild clinical disturbance, short speech samples, and careful control of task distribution. They also underscore the importance of reporting not only classification metrics but also underlying feature distributions and task composition when comparing results across studies in psychosis language research.

The task-effect analysis demonstrated that the “AboutYourself” prompt used during the interview (for data collection) consistently produced lower mean word similarity and higher variance than the “HowsItGoing” prompt in both SSD and control groups, with all within-participant comparisons reaching statistical significance. This indicates that connectedness measures are sensitive to differences in topic and discourse demands on the participant, even within the same clinical population. Similar task dependencies have been noted in work comparing narrative, picture description, and fluency tasks for coherence and graph-based metrics in psychosis (Nikzad et al., 2022). Methodologically, this implies that studies pooling speech from different prompts must either stratify by task, balance tasks across groups, or explicitly model task as a factor; otherwise, apparent diagnostic effects may be confounded by elicitation differences.

The diagnostic utility of repetition frequency as a complementary feature is noteworthy. WA showed higher repetition rates than SSD, consistent with the well-documented role of verbal perseveration in fluent aphasia as a consequence of impaired lexical access and failure to inhibit previously retrieved word forms within perisylvian networks (Goodglass and Kaplan, 1983, Robson et al., 2012). By contrast, word-level repetition is not a primary feature of SSD speech; sentence and discourse-level abnormalities such as derailment and tangentiality are substantially more common in thought-disordered speech than single-word anomalies (Andreasen, 1979, Kuperberg, 2010), and the disorganization characteristic of FTD manifests as loosened associative links between ideas rather than repetitive reuse of specific lexical items (Manschreck et al., 1985, Çokal et al., 2018).

### 4.2 Limitations and future directions

Several limitations should be considered when interpreting these findings. First, sample sizes per group are modest, and WA and SSD data were drawn from different corpora with inevitable differences in recruitment, recording, and transcription protocols, despite efforts to harmonize these different task types. The two clinical groups also differ substantially on several participant-level variables beyond diagnosis, including age, educational background, cognitive status, socioeconomic factors, and, in the case of SSD, psychotropic medication use. These factors may independently influence the linguistic features examined and further limit direct comparison between groups. Second, speech samples were relatively short, which constrains the reliability of distributional features and may have particularly impacted SSD-related analyses. Third, although we systematically examined syntactic and semantic features, the feature space is not exhaustive; other measures, including speech-acoustic related measures, graph-theoretic metrics, or more fine-grained pragmatic and discourse features, may capture complementary aspects of language disturbance in SSD and WA.

Future work should therefore prioritize larger, prospectively collected datasets in which aphasic and psychiatric groups are assessed under matched speech tasks and recording conditions. Combining connectedness measures with other linguistic and acoustic features, as well as with structural and functional neuroimaging, could help bridge the gap between observed language patterns and underlying brain mechanisms in WA and SSD. Finally, systematic comparisons of supervised, few-shot, and zero-shot LLM approaches, with standardized prompts and shared corpora, will be essential to determine the conditions under which foundation models can serve as reliable clinical adjuncts rather than opaque black boxes.

## CRediT authorship contribution statement

**Perry van der Zande:** Writing – original draft, Visualization, Software, Investigation, Formal analysis, Data curation. **Andreas van Cranenburgh:** Writing – review & editing, Supervision, Methodology. **Frank Tsiwah:** Writing – review & editing, Supervision, Methodology, Conceptualization.

## Funding

There is no funding to declare for this study.

## Declaration of competing interest

The authors declare that they have no known competing financial interests or personal relationships that could have appeared to influence the work reported in this paper.

## Appendix A Supplementary data

MMC S1

## Footnotes

1 The dependency relations are described at https://universaldependencies.org/u/dep/index.html.

2 For the list of POS tags, cf. https://universaldependencies.org/u/pos/index.html.

3 https://github.com/P-Zande/ssd-vs-wa.
