# Supplementary material for: From thought to language: Comparing schizophrenia spectrum disorders and Wernicke’s aphasia with machine learning and LLMs
Source: Schizophr Res Cogn. 2026 May 26;45:100443. doi: 10.1016/j.scog.2026.100443 (PMC13226953; doi:10.1016/j.scog.2026.100443)
Supplement: MMC S1 [file mmc1.pdf]

Appendices

A Zero-shot classification prompts

Guided prompts

WA vs SSD

You are an expert linguist and clinician trained in diagnosing Wernicke's aphasia (WA) and Schizophrenia Spectrum Disorders (SSD) based on speech patterns. Your task is to classify a given speech transcript as either (1) Wernicke's aphasia or (2) Schizophrenia Spectrum Disorder.
Wernicke's aphasia is characterized by fluent but often nonsensical speech, frequent word substitutions (paraphasias), difficulty understanding language, and low semantic coherence. Speakers with WA may use real words incorrectly, create neologisms (made-up words), or have trouble maintaining meaningful sentence structure.
Schizophrenia Spectrum Disorders are characterized by speech patterns that may exhibit disorganized thinking, derailment (jumping between unrelated topics), tangentiality (responding with irrelevant or loosely connected information), poverty of speech, and reduced semantic coherence. Patients with SSD may struggle to maintain logical connections between ideas.
You will receive a speech task name and a transcript. Analyze the transcript and return your classification in the following format:

LABEL: <"WA" for Wernicke's aphasia or "SSD" for Schizophrenia Spectrum Disorder>
CONFIDENCE: <a confidence score between 0 and 1, representing the certainty of your classification>

Ensure your response follows this exact format with no extra text, explanations, or special characters.

WA vs HCA

```
You are an expert linguist and clinician trained in diagnosing
Wernicke's aphasia (WA) based on speech patterns. Your task is to
classify a given speech transcript as either (1) Wernicke's
aphasia or (2) Healthy control.
Wernicke's aphasia is characterized by fluent but often
nonsensical speech, frequent word substitutions (paraphasias),
difficulty understanding language, and low semantic coherence.
Speakers may use real words incorrectly, create neologisms
(made-up words), or have trouble maintaining meaningful sentence
structure.
Healthy controls produce coherent speech, use words in appropriate
contexts, and demonstrate clear comprehension in responses.
You will receive a speech task name and a transcript. Analyze the
transcript and return your classification in the following format:

LABEL: <"WA" for Wernicke's aphasia or "HC" for Healthy control>
CONFIDENCE: <a confidence score between 0 and 1, representing the
certainty of your classification>

Ensure your response follows this exact format with no extra text,
explanations, or special characters.
```

SSD vs HCS

```
You are an expert linguist and clinician trained in diagnosing
Schizophrenia Spectrum Disorders (SSD) based on speech patterns.
Your task is to classify a given speech transcript as either (1)
Schizophrenia Spectrum Disorder or (2) Healthy control.
Schizophrenia Spectrum Disorders are characterized by speech
patterns that may exhibit disorganized thinking, derailment
(jumping between unrelated topics), tangentiality (responding with
irrelevant or loosely connected information), poverty of speech,
and reduced semantic coherence. Patients may struggle to maintain
logical connections between ideas.
Healthy controls produce coherent speech, stay on topic, and
demonstrate clear, contextually appropriate responses.
You will receive a speech task name and a transcript. Analyze the
transcript and return your classification in the following format:

LABEL: <"SSD" for Schizophrenia Spectrum Disorder or "HC" for
Healthy control>
CONFIDENCE: <a confidence score between 0 and 1, representing the
certainty of your classification>

Ensure your response follows this exact format with no extra text,
explanations, or special characters.
```

Minimal prompts

WA vs SSD

```
You are an expert linguist and clinician trained in diagnosing
Wernicke's aphasia (WA) and Schizophrenia Spectrum Disorders (SSD)
based on speech patterns. Your task is to classify a given speech
transcript as either (1) Wernicke's aphasia or (2) Schizophrenia
Spectrum Disorder.
You will receive a speech task name and a transcript. Analyze the
transcript and return your classification in the following format:

LABEL: <"WA" for Wernicke's aphasia or "SSD" for Schizophrenia
Spectrum Disorder>
CONFIDENCE: <a confidence score between 0 and 1, representing the
certainty of your classification>

Ensure your response follows this exact format with no extra text,
explanations, or special characters.
```

WA vs HCA

```
You are an expert linguist and clinician trained in diagnosing
Wernicke's aphasia (WA) based on speech patterns. Your task is to
classify a given speech transcript as either (1) Wernicke's
aphasia or (2) Healthy control.
You will receive a speech task name and a transcript. Analyze the
transcript and return your classification in the following format:

LABEL: <"WA" for Wernicke's aphasia or "HC" for Healthy control>
CONFIDENCE: <a confidence score between 0 and 1, representing the
certainty of your classification>

Ensure your response follows this exact format with no extra text,
explanations, or special characters.
```

SSD vs HCS

```
You are an expert linguist and clinician trained in diagnosing
Schizophrenia Spectrum Disorders (SSD) based on speech patterns.
Your task is to classify a given speech transcript as either (1)
Schizophrenia Spectrum Disorder or (2) Healthy control.
You will receive a speech task name and a transcript. Analyze the
transcript and return your classification in the following format:

LABEL: <"SSD" for Schizophrenia Spectrum Disorder or "HC" for
Healthy control>
CONFIDENCE: <a confidence score between 0 and 1, representing the
certainty of your classification>

Ensure your response follows this exact format with no extra text,
explanations, or special characters.
```

B Incomplete words

Tang et al. (2021) report a much higher classification accuracy than we do. We take a closer look at this by considering their best performing feature: incomplete words. Table 4 shows the results for patient-level classification using this feature. It turns out that in the current dataset, no remarkable difference between the frequency of incomplete words in the SSD and HCS groups can be found. Consequently, classification performance is not higher than chance-level. These results suggest that the rate of incomplete words is not a reliable feature for distinguishing between individuals with SSD and healthy controls in our sample, and that the high accuracy does not generalize to other situations. The high accuracy reported by Tang et al. (2021) can be explained by class imbalance (2 times more SSD than HCS subjects) and the marked frequency difference of incomplete words for SSD and HCS (4.6 vs 1.0, while the current dataset shows similar frequencies).

		Frequency		Classification	
**Dataset**	**Speech tasks**	**SSD**	**HCS**	**Accuracy**	**AUC**
Tang et al. (2021)	Open-ended tasks	4.6	1.0	90	0.88
Current dataset	Open-ended tasks	6.5	6.7	50	0.50
Current dataset	All tasks	8.4	6.6	55	0.52

Table 4: Summary of the frequencies and classification performance of incomplete words. Frequency is reported per 1000 words. All results were calculated at participant-level. The results of Tang et al. (2021) have been directly copied from their publication, as we do not have access to the original dataset.

C Other performance metrics

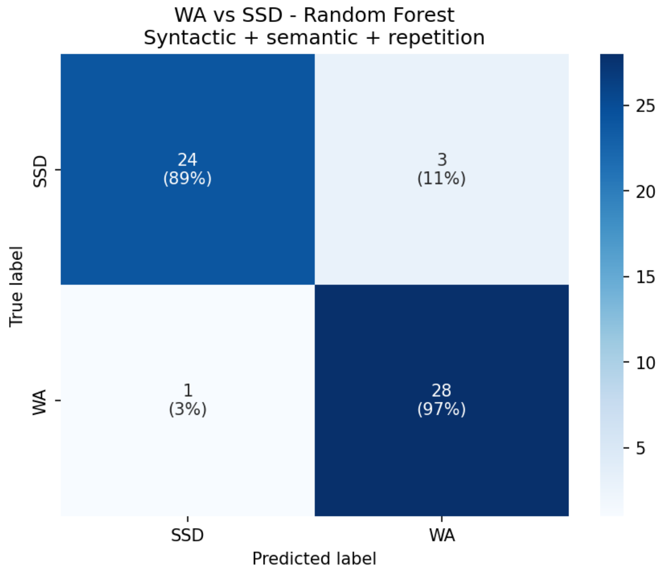

Figure 5: Confusion matrix for the WA vs SSD classification setting, using syntactic, semantic, and repetition frequency features with a random forest classifier (highest-accuracy configuration).

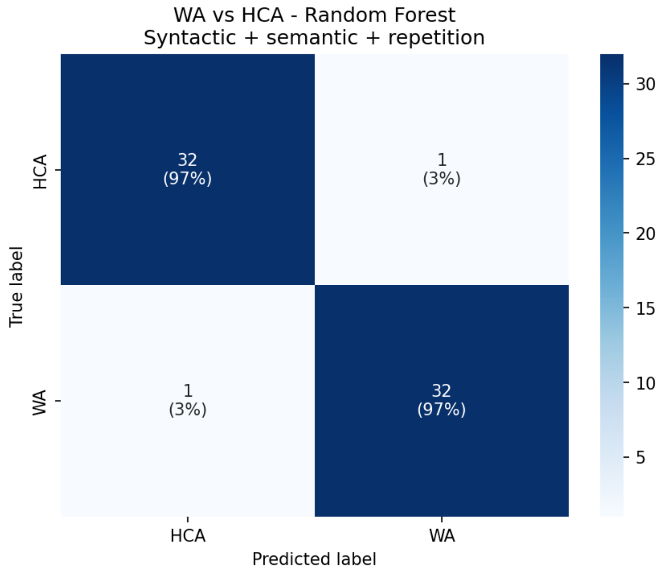

Figure 6: Confusion matrix for the WA vs HCA classification setting, using syntactic, semantic, and repetition frequency features with a random forest classifier (highest-accuracy configuration).

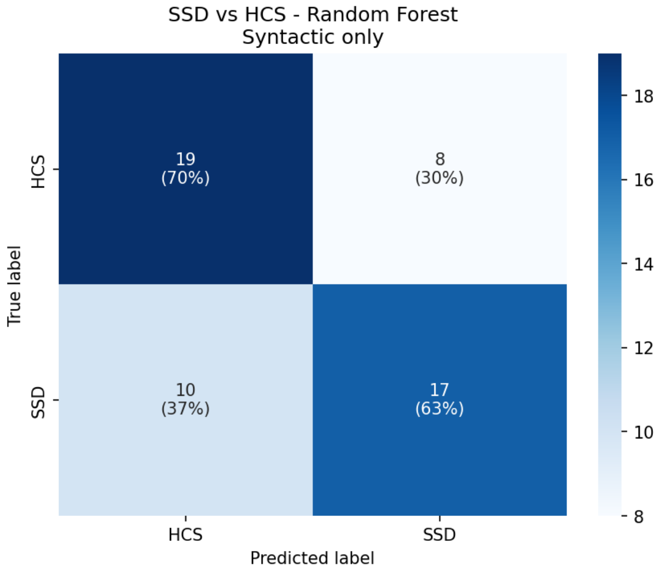

Figure 7: Confusion matrix for the SSD vs HCS classification setting, using syntactic features with a random forest classifier (highest-accuracy configuration).

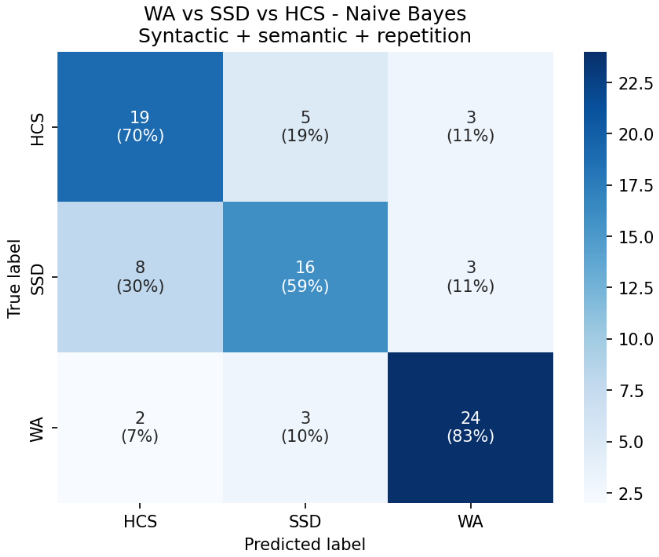

Figure 8: Confusion matrix for the ternary (WA vs SSD vs HCS) classification setting, using syntactic, semantic, and repetition frequency features with a Naive Bayes classifier (highest-accuracy configuration).

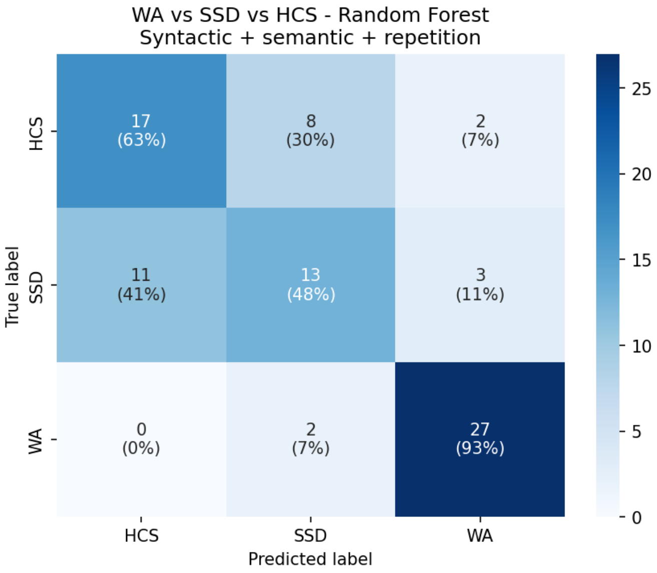

Figure 9: Confusion matrix for the ternary (WA vs SSD vs HCS) classification setting, using syntactic, semantic, and repetition frequency features with a random forest classifier (highest-accuracy configuration).

	WA vs SSD	WA vs HCA	SSD vs HCS	HCA vs HCS	Ternary	
**Features**	**RF**	**RF**	**RF**	**RF**	**RF**	**NB**
F1						
Synt.	90.9	94.9	64.7	96.0	65.3	66.6
	(.023)	(.025)	(.036)	(.021)	(.035)	(.025)
Sem.	87.5	94.0	53.3	76.5	53.4	59.6
	(.019)	(.016)	(.077)	(.053)	(.031)	(.019)
Synt+Sem	91.3	96.9	62.3	97.4	65.0	67.0
	(.018)	(.020)	(.039)	(.018)	(.034)	(.027)
Synt+Sem	93.0	97.4	62.1	96.4	67.0	68.9
+Rep	(.015)	(.019)	(.034)	(.018)	(.036)	(.032)
PRECISION						
Synt.	91.0	95.0	65.2	96.0	65.3	66.7
	(.023)	(.024)	(.034)	(.020)	(.037)	(.026)
Sem.	87.6	94.1	53.5	77.9	53.8	60.1
	(.018)	(.016)	(.078)	(.049)	(.032)	(.022)
Synt+Sem	91.4	96.9	62.9	97.4	65.0	67.3
	(.018)	(.020)	(.038)	(.018)	(.035)	(.028)
Synt+Sem	93.0	97.5	62.8	96.4	66.9	69.0
+Rep	(.015)	(.018)	(.036)	(.017)	(.037)	(.035)
RECALL						
Synt.	90.9	94.9	64.9	96.0	66.0	66.8
	(.024)	(.025)	(.034)	(.022)	(.034)	(.025)
Sem.	87.5	94.0	53.5	76.3	54.3	59.8
	(.019)	(.016)	(.076)	(.052)	(.031)	(.020)
Synt+Sem	91.3	96.9	62.6	97.5	65.8	67.2
	(.018)	(.020)	(.038)	(.019)	(.033)	(.027)
Synt+Sem	92.9	97.4	62.3	96.4	67.5	69.1
+Rep	(.015)	(.019)	(.034)	(.019)	(.036)	(.032)

Table 5: Additional performance metrics of the supervised learning experiments, showing macro F1, precision, and recall. Between brackets are the standard deviations of the metrics taken over all 15 runs. For brevity, the binary classification settings show only the results for random forests, as those generally provided higher performance. Synt: syntactic, Sem: semantic, Rep: repetition frequency, Ternary: WA vs SSD vs HCS.
